# Comparison of Low and High Temperature Sintering for Processing of Bovine Bone as Block Grafts for Oral Use: A Biological and Mechanical In Vitro Study

**DOI:** 10.3390/bioengineering10040473

**Published:** 2023-04-13

**Authors:** Asrar Elahi, Warwick Duncan, Kai-Chun Li, John Neil Waddell, Dawn Coates

**Affiliations:** Sir John Walsh Research Institute, Faculty of Dentistry, University of Otago, North Dunedin 9016, New Zealand

**Keywords:** biocompatibility, bone, physico-mechanical testing, xenograft

## Abstract

Large oral bone defects require grafting of bone blocks rather than granules to give physically robust, biocompatible and osteoconductive regeneration. Bovine bone is widely accepted as a source of clinically appropriate xenograft material. However, the manufacturing process often results in both reduced mechanical strength and biological compatibility. The aim of this study was to assess bovine bone blocks at different sintering temperatures and measure the effects on mechanical properties and biocompatibility. Bone blocks were divided into four groups; Group 1: Control (Untreated); Group 2: Initial boil for 6 h; Group 3: Boil 6 h followed by sintering at 550 °C for 6 h; Group 4: Boil 6 h followed by sintering at 1100 °C for 6 h. Samples were assessed for their purity, crystallinity, mechanical strength, surface morphology, chemical composition, biocompatibility and clinical handling properties. Statistical analysis was performed using one-way ANOVA and post-hoc Tukey’s tests for normally distributed and Friedman test for abnormally distributed quantitative data from compression tests and PrestoBlue™ metabolic activity tests. The threshold for statistical significance was set at *p* < 0.05. The results showed that higher temperature sintering (Group 4) removed all organic material (0.02% organic components and 0.02% residual organic components remained) and increased crystallinity (95.33%) compared to Groups 1–3. All test groups (Group 2–4) showed decreased mechanical strength (MPa: 4.21 ± 1.97, 3.07 ± 1.21, 5.14 ± 1.86, respectively) compared with raw bone (Group 1) (MPa: 23.22 ± 5.24, *p* <0.05), with micro-cracks seen under SEM in Groups 3 and 4. Group 4 had the highest biocompatibility (*p* < 0.05) with osteoblasts as compared to Group 3 at all time points in vitro. Clinical handling tests indicated that Group 4 samples could better withstand drilling and screw placement but still demonstrated brittleness compared to Group 1. Hence, bovine bone blocks sintered at 1100 °C for 6 h resulted in highly pure bone with acceptable mechanical strength and clinical handling, suggesting it is a viable option as a block grafting material.

## 1. Introduction

Worldwide, approximately 2.2 million people undergo bone grafting procedures annually in orthopedics, neurosurgery and dentistry [1]. In dentistry, oral bone loss occurs following periodontal diseases, maxillofacial trauma or pathology and tooth extraction [2]. Treatment often involves strategies to enhance regeneration of the missing bone using bone grafting materials [3,4], with the most common being autografts, allografts, xenografts and synthetic bone substitutes [5].

Autografts, obtained from a secondary site in the patient, remain the gold standard because of their osteoconductive, osteoinductive and osteogenic properties [6,7,8]. Disadvantages of such autologous bone block grafts are their limited availability and donor site morbidity [9]. Allografts are usually harvested from live or deceased donors and have been associated with patient and therapist reservations because of a previously described risk of infection [10,11,12]. Xenografts are often obtained from animal sources such as bovine bone. Due to their animal origin, xenografts have similar bone quality as humans and avoid donor-site morbidity but carry the risk of disease or infection transmission [11,12]. To minimize viral and bacterial contamination, bone is processed and sterilized to remove organic material and unwanted antigens [4].

Several methods for the processing and sterilization of bone grafts have been used, including boiling, sintering, freeze-drying, gamma irradiation and autoclaving, all of which can adversely affect the biological properties and mechanical strength of the graft [13,14,15]. Consequences can include graft fragility and poor handling qualities as well as reduced induction/conduction of bone regeneration within the healing surgical site.

Moreover, not all processing methods result in the same level of organic removal. Commercially available and commonly used block bone grafts have been assessed histologically for organic or cellular remnants [16]. Three out of the five commercial bone blocks contained histologically observable organic/cellular remnants, despite the products stating that their blocks were free of such remnants. Such remnants could result in undesired immune responses, compromising safety, biocompatibility and function [16].

One method of purification and processing of bone grafts is high-temperature sintering at 300–1300 °C to completely remove zoonotic infectious and immunogenic agents present in the bone via thermal decomposition, allowing use in humans [17]. Sintered bovine bone was first used by Ueno et al. in 1982 as a substitute for new bone [18,19,20]. This material was named a true bone ceramic because of its bone and ceramic-like characteristics.

High-temperature sintering combusts the intrinsic collagen components in the bone and alters the native porous and crystalline structures. This technique converts the native bone minerals into inorganic hydroxyapatite and tri-calcium phosphate, which is much less bioabsorbable relative to native bone but helps retain some mechanical strength [17,21,22,23].

Bone grafts in block form are often needed in clinical practice for the reconstruction of larger bony defects. However, bone processing often compromises handing properties, along with mechanical and biological compatibility. During surgery, block grafts are rigidly fixed onto the bone bed with screws that are placed using controlled speed and torque. The processed block grafts must have sufficient strength to withstand such clinical manipulation. Gherke et al., 2019, compared sintered bovine bone blocks with chemically purified bone blocks and found no statistically significant difference in the mechanical strength of the two groups; however, both groups showed low cell viability [24,25]. This suggests that sintering may not be the optimum processing method and requires further development.

The thermal decomposition of bone has been categorized into various phases. Phase 1 is between 0 °C and 200 °C, in which dehydration occurs. Phase 2 is characterized by decomposition of the collagen and lipids, which occurs between 200 °C and 900 °C; the final carbonate decomposition/inversion phase occurs beyond 900 °C [26]. The pretreatment and temperature at which sintering is conducted determines the thermal decomposition of the bone blocks and subsequently determines the physico-chemical and biological properties of the blocks.

Various sintering temperatures and pre- or post-heating steps have been tested for producing grafts. A pioneering study on sintered bovine bone conducted by Ueno et al. (1983), who sintered at 600 °C followed by 1100 °C/1450 °C after boiling in water and chemically deproteinizing the bone [20]. Their constructs were tested in a rabbit model as well as human clinical trials, and they claimed good integration with new bone formation [20]. Boiling as a pre-treatment step is often used to avoid soot and crack formation during treatment as well as removal of marrow and soft tissue residues [27,28]. Another study conducted on bovine cancellous bone samples assessed three treatments: 300 °C for 3 h (Group 1), 300 °C for 3 h followed by 530 °C for 6 h (Group 2) and 300 °C for 3 h followed by 1000 °C for 6 h, with a degreasing and deproteinization step prior to the sintering (Group 3) [15]. Group 1 and 2 showed the best cellular attraction and enhanced the differentiation of MSCs. Moreover, testing in an in vivo rabbit cranial model supported the in vitro results. Another study tested sintering of bovine cancellous bone at 1120 °C (3 h) and 1350 °C (5 h) with 30% hydrogen peroxide and ethanol pre-treatment stages [29]. These treatments both increased osteoblast metabolic as well as alkaline phosphatase activity. None of these studies tested bone ‘block’ graft preparation based on sintering. Moreover, to the best of our knowledge, no study has investigated the effect of sintering at 550 °C (6 h) and 1100 °C (6 h) after only boiling without any chemical treatment, while assessing both mechanical and biological acceptability of a bovine bone block graft for oral grafting.

The objective of this study was to investigate the effect on bovine bone blocks of two different (low and high) temperature sinterings, consisting of boiling for 6 h followed by sintering at 550 °C or boiling for 6 h followed by sintering at 1100 °C. Bone block purification, crystallinity, mechanical strength and biocompatibility were compared to untreated bone blocks.

## 2. Materials and Methods

### 2.1. Bone Sample Preparation

Fresh bovine femur condyles were sourced from a local butcher and sectioned in a cutting machine (Accutom-50, Struers, Ballerup Denmark) using a diamond cutting disc (MOD13, Struers, Ballerup, Denmark) with a feed speed of 0.120 mm/s at 3200 rpm. For thermogravimetric analysis (TGA), mechanical testing and X-Ray diffraction (XRD) analysis, blocks were produced with final dimensions of 5 × 5 × 5 mm^3^; for the proliferation assay and phalloidin/DAPI staining, the block dimensions were 20 × 20 × 2 mm^3^.

After optimizing boiling and sintering times (Supplementary Figures S1 and S2), four groups were selected for full investigation. These were:

1. Group 1: Control (untreated bone);
2. Group 2: boiled for 6 h in a pressure multi-cooker (Crockpot, Model: CPE300, Boca Raton, FL, USA) with distilled H_2_O covering the specimens at a volume of 10 mL/mg bone and renewed every 2 h;
3. Group 3: boiled for 6 h and then sintered in a dental furnace (MESTRA^®^, Txorierri Etorbidea, Spain) at 550 °C for 6 h;
4. Group 4: boiled for 6 h and then sintered at 1100 °C for 6 h.

### 2.2. Residual Organic Content Analysis Using Thermogravimetric Analysis

A Q50 Thermogravimetric Analyzer (TA instruments, New Castle, DE, USA) was used to determine the residual organic content in all the treated bone samples as well as controls (weight precision: +/− 0.01%, sensitivity: 0.1 µg, isothermal temperature accuracy: +/− 1 °C and isothermal temperature precision: +/− 0.1 °C). TGA was conducted at a heating rate of 20 °C per minute, from 20 °C to 1000 °C. Mass loss was analyzed using Advantage/Universal Analysis (UA) software (version 5.5.24) and interpreted based on the associated temperature ranges. Up to 200 °C was attributed to loss of evaporated and bound water, 200–375 °C was considered loss of organic tissue due to decomposition, 375–550 °C was loss of residual organic tissue due to combustion and 550–775 °C was characterized as carbonate decomposition [26,30].

### 2.3. Mechanical Strength Using Compression Testing

Compression testing was conducted on 5 × 5 × 5 mm^3^ bone blocks prepared from the femur condyles of the same animal. Initially, the first phase of thermal treatment (boiling) was optimized using compression testing (*n* = 30) to investigate the effects of pre-boiling of the bone for different durations. The samples were pre-boiled for 2, 6, 12 and 18 h and then subjected to sintering at 1100 °C for 3 h. We optimized the second thermal treatment phase (sintering) by investigating different durations of sintering at 1100 °C after a 6 h pre-boil. The samples were subjected to sintering at 3 h, 6 h and 9 h.

Control and experimental bone samples (*N* = 30 samples per group) were tested with a universal testing machine (Instron 3369, Instron, Norwood, MA, USA) equipped with a 500 N (for group 2, 3 and 4) and 1000 N (for group 1) load cell at a crosshead speed of 2 mm/min (±1% of reading from 1/200 to 1/500 of the load cell capacity). Bluehill Universal software (Instron Corporation, Norwood, MA, USA) was used during testing to record and calculate the compressive stress at maximum load (MPa).

### 2.4. Crystallinity Analysis of Bone Blocks

Powdered X-ray diffraction (XRD; PANalytical X’Pert Pro MPD system, Malvern Panalytical, Malvern, UK) was used to determine the degree of crystallinity of the treated bone samples and controls semi-quantitatively (long range accuracy: ±0.0025°; short range (0.5°) accuracy: ±0.0004°; and angular reproducibility: <0.0002°). The scans were performed at 5–80° angle ranges; degree of crystallinity was calculated using OriginPro 2008 software (Origin Lab Corporation, Northampton, MA, USA) by subtracting the crystalline peaks from the total area of the graph (i.e., crystalline and amorphous area). We used the peak analyzer function of the software to locate all peaks and multiple peak fit, with the Gaussian peak fit function to separate overlapping peaks [31,32].

### 2.5. Bone Microstructure Using Scanning Electron Microscopy and Chemical Characterization Using Energy Dispersive Spectroscopy 

The microstructure of each group (*n* = 2) was assessed using a scanning electron microscope (SEM) equipped with energy-dispersive spectroscopy (EDS) (Zeiss Sigma VP FEG SEM, JEOL FE-SEM 6700, Joel Ltd., Tokyo, Japan). The electron beams were set to an accelerating voltage of 10 kV. Samples were gold-palladium coated (for SEM) or carbon coated (for EDS). Samples were viewed at 70×, 1000× and 10,000× magnifications. Photomicrographs were correlated in Adobe Photoshop (version 23.1.0; Adobe Systems, San Jose, CA, USA).

### 2.6. Qualitative Mechanical Assessment of Blocks Using a Drill Test

To test the feasibility of using the graft blocks (Groups 1–4) in a clinical setting, bone blocks measuring 5 × 5 × 5 mm^3^ (*n* = 2 per group) were drilled at 800 rpm using successively two latch type drill bits of 1.1 mm and 1.5 mm diameter in a dental implant handpiece and surgical motor (Implant MED, W&H, Bürmoos, Austria). A 10 mm long × 1.5 mm diameter titanium block fixation screw (Salvin, Charlotte, NC, USA) was screwed through the bone block at 10 Ncm insertion using the surgical motor and driver, and finalized with a manual screwdriver (Straumann AG, Basel, Switzerland) as required. The bone blocks were fixed on a wooden block, which acted as the recipient bone bed site. The blocks were qualitatively assessed for any cracks or fracture during drilling and screw fixation.

### 2.7. Biological Validation

#### 2.7.1. Human Calvarial Osteoblast (HCO) Cell Culture

Human Calvarial Osteoblasts (HCOs) (Cat. No. 4600, lot 3439, ScienCell Research Laboratories, Carlsbad, CA, USA) were cultured at 37 °C in a humidified atmosphere of 95% air and 5% CO_2_ in osteogenic media comprising DMEM with GlutaMax (Cat. No. 10566032, ThermoFisher Scientific, Waltham, MA, USA), supplemented with 10% fetal bovine serum (FBS; Cat. No. 10091148, Invitrogen, ThermoFisher Scientific, Waltham, MA USA), 1% antibiotic-antimycotic (Penicillin (100 unit/mL), streptomycin (100 µg/mL), amphotericin B (250 ng/mL)), 0.5% gentamycin (50 µg/mL) and osteogenic supplements of 155.2 µM L-ascorbic acid 2-Phosphate (Cat. No. 49752, Sigma, Darmstadt, Germany) and 10 nM dexamethasone (Cat. No. D2915, Sigma, Darmstadt, Germany). Media was renewed every 48 h. HCOs were cultured from passage 6 to passage 8 and then cryopreserved using 10% dimethyl sulfoxide (DMSO). HCOs were thawed and expanded before seeding on bone blocks.

#### 2.7.2. HCO Metabolic Activity Assessment (PrestoBlue™)

Four bone slices (20 × 20 × 2 mm^3^) from each group were punched into 5 mm diameter discs using a 5.2 mm circular soft tissue punch (Cat. No. 32Z2002 Nobel Biocare, Kloten, Switzerland). Bone samples were sterilized in 70% ethanol for 10 min thrice and washed twice with PBS. Four bone samples per group were seeded with cells, and one bone sample without cells was kept as a control. Three empty wells with media only were reserved for control assays. Assays were conducted in 96-well plates (Falcon, Becton Dickinson Labware, Franklin Lakes, NJ, USA), with bone samples incubated with osteogenic media for 24 h prior to seeding with HCOs. HCOs were seeded at 1.2 × 10^4^ cells per sample in 200 μL of osteogenic media and cultured at 37 °C in a humidified atmosphere of 95% air and 5% CO_2_. After 6 h, bone samples were shifted to a new plate with 200 μL of new osteogenic media. Serum starvation (osteogenic media containing only 5% FBS) was conducted for 18 h to synchronize the cell cycle, after which osteogenic medium with 10% FBS was added. The metabolic activity of HCOs on bone samples was measured at 24, 48, 72 and 96 h of culture by adding 10% PrestoBlue™ to the medium 4 h prior to each time point. After incubation, the medium was aspirated and aliquoted into a new 96-well plate, and fresh osteogenic medium was added to the samples. Fluorescence was measured at an excitation/emission of 560/590 nm in a multi-well plate reader (Synergy 2, Biotek, Shoreline, WA, USA) (accuracy specification of ±1.0% ± 0.010 OD or better and a repeatability specification of ±1.0% ± 0.005 OD or better). The experiment was conducted in quadruplicate, and the control (medium with bone sample only, without cells) was subtracted from each groups’ fluorescence units prior to graphing in GraphPad PRISM Version 9.3.1 (350) (San Diego, CA, USA).

#### 2.7.3. Observation of Cellular Adhesion by Actin Filaments’ Staining Using Phalloidin and Nuclei Staining Using DAPI

Bone discs of 5 mm diameter and 2 mm thickness from Groups 1–4 were investigated (*n* = 4). To observe the actin cytoskeleton of osteoblasts attached to the bone surfaces, HCOs were seeded at 1.2 × 10^4^ cells per disc in a 96-well plate. To ensure maximum cells were seeded on the bone and not on the plastic well, cells were suspended in a 25 µL drop of osteogenic media, placed on top of the discs and incubated for 4 h, after which 200 µL of osteogenic media was added. After 48 h, the samples were fixed in neutral buffered 3.7% methanol-free formaldehyde solution (Cat. No. 1.04003.2500, Merck, Darmstadt, Germany) for 15 min at RT. Samples were washed with PBS thrice and permeabilized in 0.1% Triton X-100 (Cat. No. T8787, Sigma-Aldrich, Darmstadt, Germany) in PBS for 15 min prior to staining with AlexaFluor 467 phalloidin (Cat. No A222287, Thermofisher Scientific, Waltham, MA, USA) for 45 min. Phalloidin stock solution was prepared using the manufacturer’s recommended DMSO stock solution methodology. Briefly, AlexaFluor 467 phalloidin was dissolved in 150 μL anhydrous DMSO to make 400× stock solution. Before staining, the stock solution was diluted by dissolving 0.5 μL of the stock solution in 200 μL of PBS for each coverslip to be stained. DAPI (4′,6-diamidino-2-phenylindole, dihydrochloride; Cat. No. D1306, ThermoFisher, Waltham, MA, USA) was used to counterstain the samples. Images were analyzed using an EVOS M5000 fluorescence microscope (Cat.No. AMF5000, ThermoFisher, Waltham, MA, USA).

### 2.8. Statistical Analysis 

Statistical analysis was performed using GraphPad PRISM software (Version 9.3.1 350) (San Diego, CA, USA). Variable distribution was evaluated by the D’Agostino–Pearson test and Shapiro–Wilk test. Levene’s test was used to assess homogeneity of variance. Tukey’s multiple comparison test based on one-way ANOVA was used for normally distributed quantitative data. For values not normally distributed, the Friedman test was performed. Differences were considered statistically significant at *p*-value < 0.05.

## 3. Results

### 3.1. Thermogravimetric Analysis to Assess Organic Content and Carbonate

**Figure 1:**
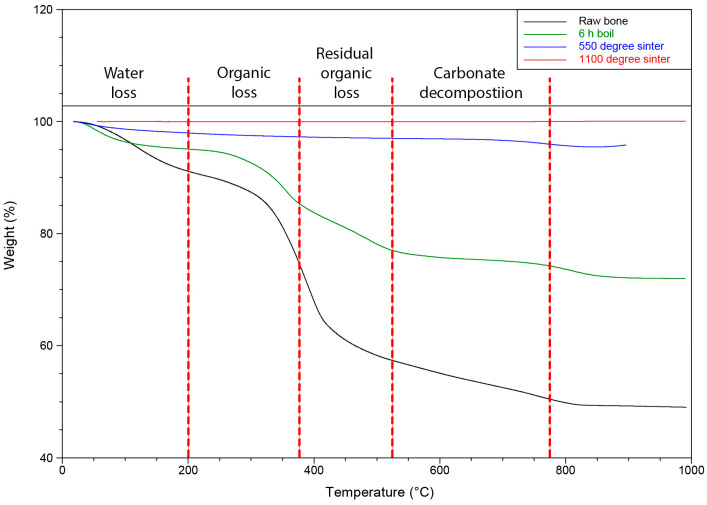
Overlay graph of TGA curves of the different groups of bovine bone blocks showing percentage weight loss with increase in temperature. Up to 200 °C = loss of evaporated and bound water, 200–375 °C = loss of organic tissue, 375–550 °C = loss of residual organic tissue and 550–775 °C = carbonate decomposition.

TGA analysis of the organic content (Figure 1, Table 1) revealed that Group 1 raw bone samples contained 17.55 wt% and 11.87 wt% of organic content and residual organic content, respectively. Boiling for 6 h (Group 2) resulted in marked reduction in organic and residual organic material, and sintering further reduced this to very low levels (Groups 3 and 4). Group 4 showed minimum organic content (0.02 wt%) and residual organic contents (0.02 wt%), indicating the greatest organic loss as compared to all the groups. In raw bone, 3.03% carbonate was present in the hydroxyapatite of samples, whereas it was decomposed to 0.96% in Group 3 and 0% in Group 4, showing the loss of carbonate from the hydroxyapatite in highly sintered bone samples.

### 3.2. Compression Strength Was Increased with High Temperature Sintering Compared to Lower Temperature Sintering

Initially, experiments were conducted to investigate the effects of different durations of pre-boiling and sintering of the bone on its mechanical strength (Supplementary Figures S1 and S2; Tables S1 and S2). Due to the lack of significant difference between different boiling and sintering durations and being more time efficient, we selected the 6 h boil followed by 6 h sintering for the study. 

**Figure 2:**
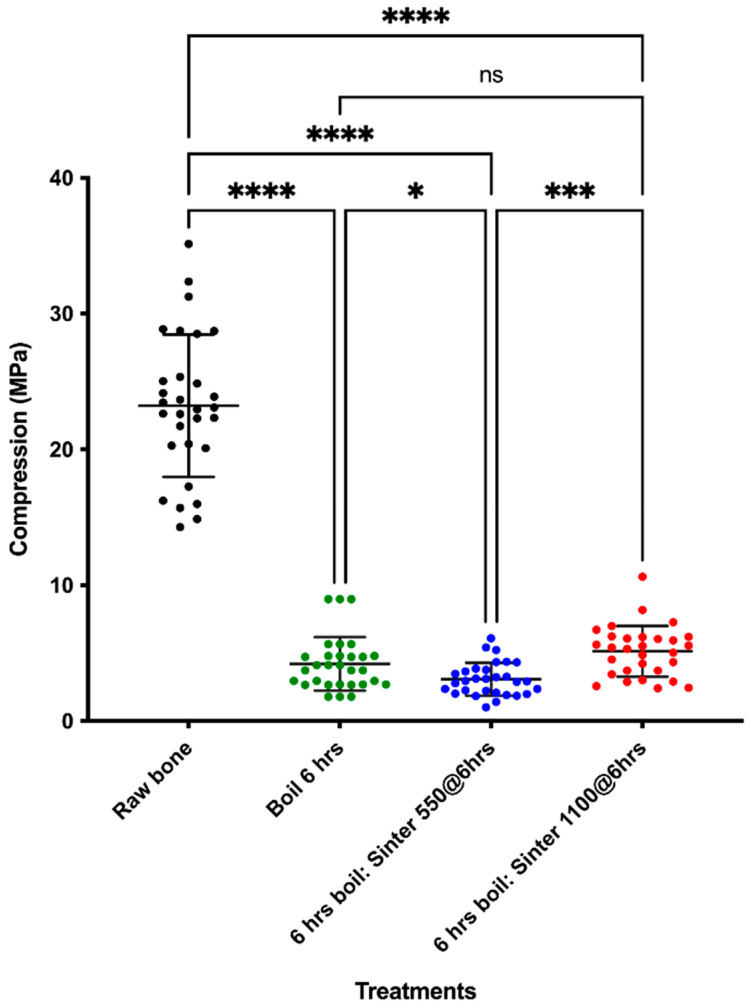
Effect of different sintering temperatures on the mechanical strength of bovine cancellous bone after boiling for six hours (*n* = 30). Mean ± SD; * *p*-value < 0.05, *** *p*-value < 0.0005, **** *p*-value < 0.0001, ns = not significant.

There was a significant reduction of mechanical strength of all treated bone block groups (Groups 2–4) (MPa: 4.21 ± 1.97, 3.07 ± 1.21 and 5.14 ± 1.86, respectively) as compared to Group 1 (raw bone) (MPa: 23.22 ± 5.24, *p* < 0.05). While the 550 °C sintered bone group (Group 3) had significantly lower mechanical strength than the 6 h boil group (Group 2), the 1100 °C sintered bone group (MPa: 5.14 ± 1.86) had significantly higher compressive strength than Groups 2 and 3 (MPa: 4.21 ± 1.97 and 3.07 ± 1.22, *p* < 0.0001, respectively) (Figure 2).

### 3.3. Crystallinity Increased with Higher Temperature Sintering

**Figure 3:**
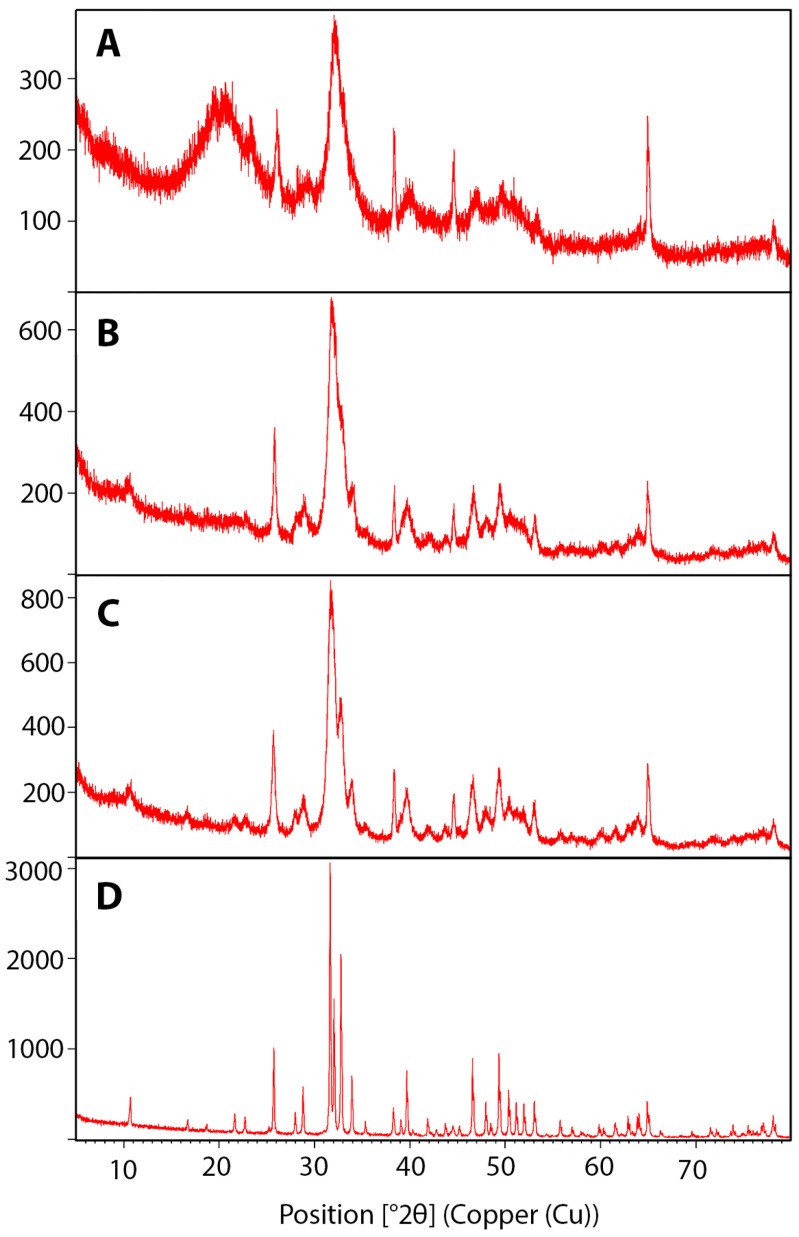
X-ray diffraction spectra of the different groups of bovine bone blocks. (**A**) Raw bone, (**B**) boil (6 h), (**C**) initial boiling (6 h) followed by 550 °C sintering (6 h), (**D**) initial boiling (6 h) followed by 1100 °C sintering (6 h).

X-ray diffraction showed that the crystallinity of bone samples increased as the processing temperature increased. Group 1 had the lowest crystallinity of 23.31%, which doubled to 48.20% after boiling. Bone sintered at the highest temperature of 1100 °C showed the highest crystallinity (95.33%) (Figure 3, Table 2).

### 3.4. Microcracks Were Detected by Scanning Electron Microscopy after Sintering

**Figure 4:**
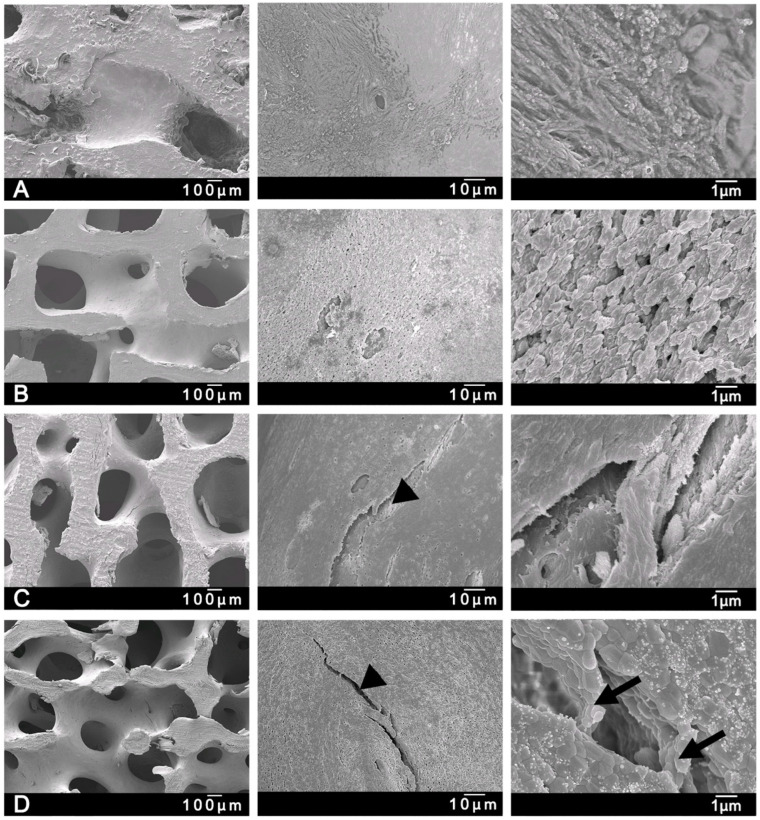
SEM micrographs of the different groups of bovine bone blocks. (**A**) Raw bone, (**B**) boil (6 h), (**C**) initial boiling (6 h) followed by 550 °C sintering (6 h), (**D**) initial boiling (6 h) followed by 1100 °C sintering (6 h). Arrows show crystal structures; arrow heads show microcracks (representative image of *n* = 2).

SEM was conducted to investigate the surface structure of the bone in the four groups (Figure 4). Bone from Group 1 samples had an abundant covering of residual cells and connective tissue on the trabecular frameworks (Figure 4A). Group 2 showed evidence of a globular surface and collagen-like fibers, which indicated that boiling did not completely remove the extracellular matrix (ECM) (Figure 4B). In Groups 3 and 4, the structural detail of trabecular pores was clearly discernible, with no evidence of any residual intertrabecular contents or debris. Due to the presence of collagen-like material, Groups 1, 2 and 3 showed structural differences to Group 4, which had a more crystalline structure. Moreover, the crystalline structures in Group 4 were visible at higher magnifications (Figure 4D). Some indications of structural deterioration, such as microcracks, were found in Groups 3 and 4 (Figure 4C,D); however, no microcracks were evident in Groups 1 and 2.

### 3.5. Chemical Characterization Using Energy Dispersive X-ray Spectroscopy 

Chemical composition of the samples obtained from random points on the surface of the samples by means of EDS are shown in Table 3 (*n* = 3). EDS analysis revealed that the Ca/P ratio of Group 2 was higher (2.2 ± 0.1, mean ± SD) than the control (1.9 ± 0.1) and Group 3/4 (2.1 ± 0.1). There was a decline in the carbon (C) values as the higher temperature treatment was applied. The calcium (Ca) and phosphorous (P) content in Groups 3 and 4 was higher than in Groups 1 and 2. Oxygen (O), P_2_O_5_ and calcium oxide (CaO) also increased as the temperature increased. Only traces of sodium, magnesium and aluminum were detected (Table 3).

### 3.6. Drill Test

**Figure 5:**
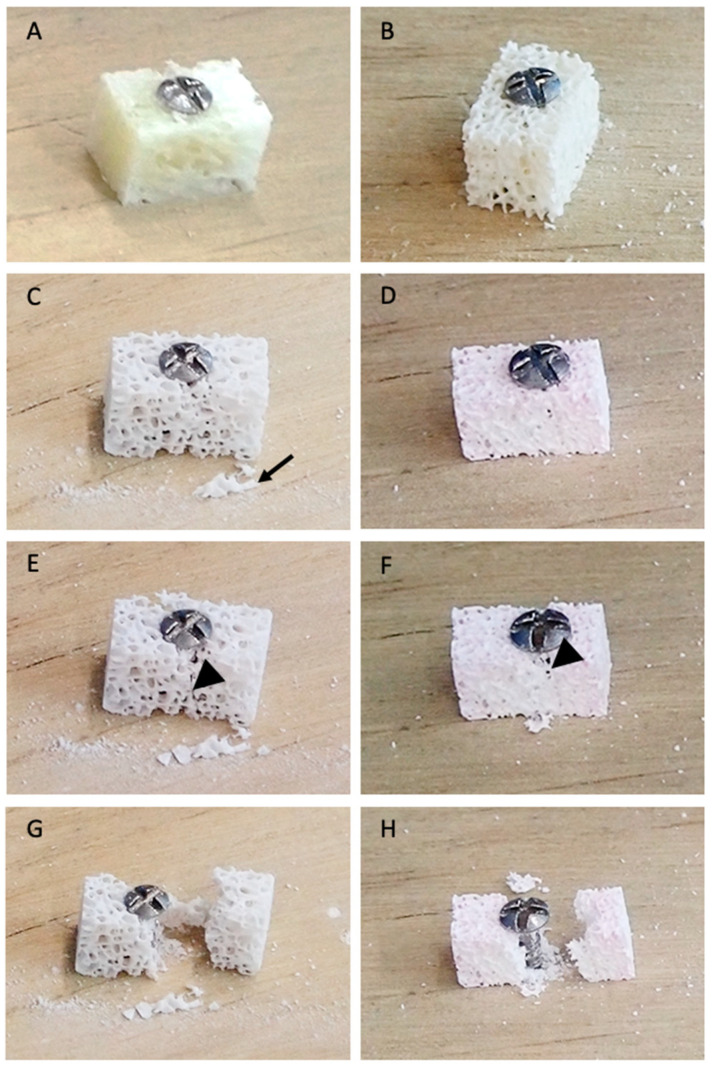
Testing the ability of the bone block to withstand fracture during placement of a bone screw. (**A**) Raw bone, (**B**) boil (6 h), (**C**,**E**,**G**) initial boiling (6 h) followed by 550 °C sintering (6 h), (**D**,**F**,**H**) initial boiling (6 h) followed by 1100 °C sintering (6 h). (**C**) showed more brittleness in the form of bone fragment chipping; (**E**,**F**) showed crack formation during screw placement; and (**G**,**H**) showed bone fracture during excess screw tightening. Arrow = chipped bone fragment; arrowhead = cracks (representative image of *n* = 2).

The simulated clinical drill test showed that Group 3 bone blocks were more brittle, as shown by the bone particles chipped off from the block as compared to Groups 1, 2 and 4 (Figure 5C). All samples were able to maintain their structures during drilling and screw placement, as long as the screw head was above the bone block surface. However, when the head of the screw was further tightened down onto the bone block, Group 3 and 4 bone block samples fractured (Figure 5G,H).

### 3.7. Metabolic Activity

**Figure 6:**
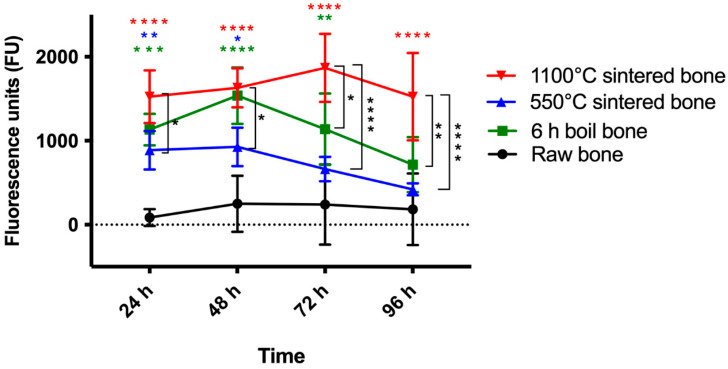
Human calvarial osteoblast (HCO) cells seeded on different treated bones assessed with PrestoBlue™ proliferation assay (*n* = 4, mean ± SD). * *p*-value ≤ 0.05, ** *p*-value ≤ 0.005, *** *p*-value ≤ 0.0005, **** *p*-value ≤ 0.0001 compared to Group 1 (raw bone) mean.

Metabolic activity assay results at 24 h, 48 h, 72 h and 96 h of HCO culture on the control as well as experimental bone discs are shown in Figure 6. Cells on Group 4 graft material had significantly higher metabolic activity compared to Group 1 from 24 h to 96 h (*p* < 0.05). Group 4 HCOs also showed significantly higher metabolic activity than Group 3 at all time points (*p* < 0.05). HCO metabolic activity on Group 2 bone was higher than Group 3 bone throughout the 4 days; however, both showed consistently decreased metabolic activity after 48 h.

### 3.8. Observing Cellular Adhesion by Phalloidin Staining

**Figure 7:**
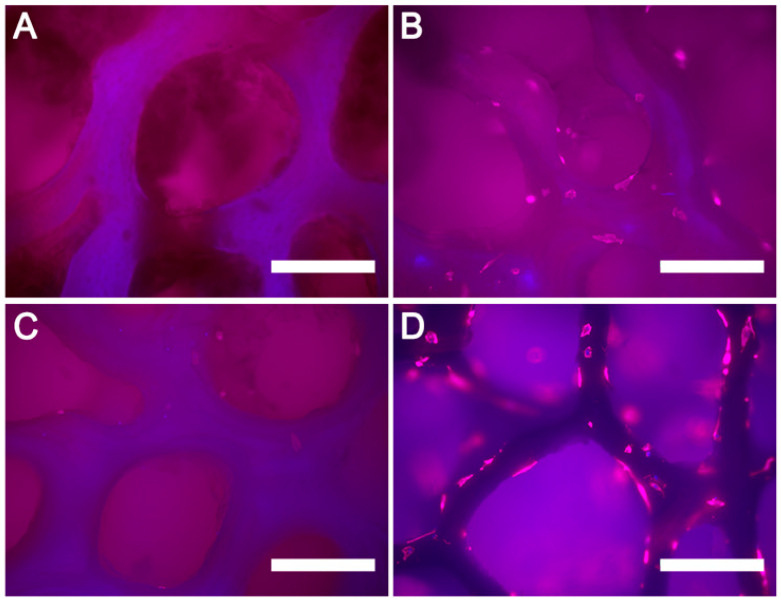
Cells attached on different treated bovine bone blocks assessed through phalloidin staining. (**A**) Raw bone, (**B**) boil (6 h), (**C**) initial boiling (6 h) followed by 550 °C sintering (6 h), (**D**) initial boiling (6 h) followed by 1100 °C sintering (6 h). Scale bar = 1050 µm (representative images of *n* = 3) (Phalloidin = red, DAPI = blue).

Adhesion to the bone surface was observed with phalloidin/DAPI immunofluorescence (Figure 7). The morphology of osteoblasts was examined after culture for 48 h on the surfaces of the control and Group 2, 3 and 4 samples. There were no cells visible on the control (raw bone) (Figure 7A), while few cells were observed attached to the Group 3 sample (Figure 7C). Interestingly, osteoblasts had spread well, and actin filaments were clearly observed in Group 2 (Figure 7B) and 4 (Figure 7D) samples. Only a single layer of cells was observed, with a clear space between the cells.

## 4. Discussion

Xenografts are a rich resource for bone block grafting. However, the process for preparation of these bone blocks must conserve the mechanical strength of the blocks for clinical use and should result in a product that is biocompatible and that promotes osteointegration. Thus, the final bone graft should be sterile and have low crystallinity, low immunogenicity, satisfactory mechanical strength, ease of handling and biocompatibility. Bone processing can have detrimental effects on the quality of bone grafts in terms of mechanical, biological and osteogenic effects. While bone processing is necessary, a reasonable balance needs to be met where sufficient purification takes place to produce sterile and safe material for use, while preserving the strength, biocompatibility and osteogenic effects of the bone. In this study, New Zealand–sourced prion-free bovine bone blocks were processed at two sintering temperatures to produce a block xenograft, with the aim of reducing the immunogenic organic contents while retaining mechanical strength and biocompatibility for human grafting.

In terms of reducing the chance of an immunological reaction, bone block processing plays a vital role in preparation. While traditional bone processing steps included delipidation and deproteinization steps, aiming to remove the various constituents of the bone grafts, our study divided the processing into two temperature ranges, based on partial organic component removal (550 °C sintering) and complete organic content removal (1100 °C sintering). Pioneering studies of sintered bone graft preparation tested sintering at 600 °C followed by 1100 °C and 1450 °C [18,20] and claimed some success in an animal model as well as clinical trials. Another study on bovine cancellous bone samples assessed sintering at 300 °C for 3 h, or 300 °C for 3 h followed by 530 °C for 6 h, or at 300 °C for 3 h followed by 1000 °C for 6 h, as well as with a degreasing and deproteinization step before the sintering [15]. This study demonstrated that the groups sintered at 530 °C or lower showed better cellular attraction and differentiation of MSCs as well as bone regeneration in a rabbit cranial model. Another study tested the sintering of bovine cancellous bone at 1120 °C (3 h) and 1350 °C (5 h) with a 30% hydrogen peroxide and ethanol pre-treatment stage [29]. Both treatments showed increased osteoblast metabolic as well as alkaline phosphatase activity. However, to the best of our knowledge, no study has tested the effect of sintering at 550 °C (6 h) and 1100 °C (6 h) after boiling alone for 6 h and assessed both mechanical and biological acceptability.

In our study, Group 3 (sintered at 550 °C) and Group 4 (sintered at 1100 °C) had lower organic content than the raw bone. These results are similar to another study that tested two commercially available graft materials processed at 300 °C (Bio-Oss^®^) and 950 °C (Gen-Ox^®^). The organic content present in Bio-Oss^®^ was 2.6% (TGA measured between 30 °C to 420 °C) and 0.5% (TGA measured between 420 °C to 635 °C) as compared to Gen-Ox^®^, which had 0.35% organic content [33]. 

Compression is the most common type of stress in vivo for bone blocks, especially during fixation with bone screws or under normal mastication load if the block is used as an onlay near a dentulous site. When stress is excessive, it can result in fracture of the bone blocks. From a mechanical point of view, the ultimate ability of a bone block to withstand compression is very important when determining its suitability as a block graft material [34]. Our initial optimization of boiling duration showed that 6 h was best for maximizing strength during the cleaning phase. We also showed that sintering for 6 h produced the best results. Based on these optimizations, we tested our samples after boiling for 6 h and sintering for 6 h at two different temperatures. Our study showed that Groups 2, 3 and 4 had reduced strength as compared to Group 1 (raw bone). Sintering bone grafts at 1100 °C significantly improved compressive strength as compared to the 550 °C treated bone. Karacayli et al. (2009) sintered fresh sheep bones at 850 °C, milled them into powder, and then compacted the material into a cylinder and sintered at 1100 °C, 1200 °C and 1300 °C and tested for compression strength [14]. They observed that 1100 °C sintered bone had a compressive strength of 52.9 MPa, while greater strength was demonstrated by the 1300 °C samples. In our study, the mean compressive strength was 5.14 ± 1.86 MPa for 1100 °C, with a maximum reading of 10.62 MPa. There was a significant reduction of strength when compared with raw bone. This may be explained by a study that compared the compressive strength of sintered bone (600 °C for 6 h) with supercritical treatment (for 12 h at 50 °C), which preserved the collagen in the bone. The authors found that the sintered bone mechanical strength was lower than that of the other groups, which they believed to be the result of loss of mineral and structural damage caused by the intensive heating. The trabecular bone matrix consists mainly of type I collagen fibers and noncollagenous proteins [35]. Noncollagenous proteins represent approximately 90% of the organic composition of the entire bone tissue and play an important role in its structural and mechanical stability. Over-treatment by sintering or chemical reagents can severely damage the structure of type I collagen fibers, as demonstrated by the grooves on the surface of the trabeculae and by the decrease in trabecular thickness. Without the support of collagen fibers, the adherent hydroxyapatite crystals can become brittle, and the mechanical strength of the granules can deteriorate [36].

X-ray diffraction showed that the crystallinity of bone samples increased as the temperature was increased. It has been established that the relatively high crystallinity delays the resorption rate of the hydroxyapatite (Hap)-a process as determined by giant cells and macrophages [37]. In Group 1 (raw bone, untreated), bone crystallinity was 23.31%; for the sintered bone Groups 3 (550 °C) and 4 (1100 °C), crystallinity was 63.15% and 95.33%, respectively. Gehrke et al. (2019) recorded the crystallinity of their 950 °C sintered bovine bone at 41%, which is half of our bone’s crystallinity when treated at 1100 °C. In another study by Pripatnanont et al. (2007), it was demonstrated that as the sintering temperature rose from 800 °C to 1200 °C, the hydroxyapatite microstructure size grew, and the surface became denser [38], which was similar to our observation during morphological analysis using SEM.

The morphological analysis of our bone block surfaces showed more residual cells and connective tissue in the trabecular meshwork of Group 1 samples along with the presence of collagen on the bone surface. After boiling alone, Group 2 samples showed evidence of a globular surface and collagen-like fibers, which indicated that boiling did not completely remove the extracellular matrix (ECM). Similar globular appearance was described in a study with sintered hydroxyapatite [39]. Sintering in Groups 3 and 4 resulted in the trabecular pores being more evident, with no signs of debris. Although ECM structure was still seen on the surface of Group 3, Group 4 showed no signs of any structures consistent with ECM. The bone surface of Group 4, however, presented with large crystal structures in its architecture, which were similar to the hydroxyapatite crystals described on the surface of deproteinized bovine xenografts in a study by Accorsi-Mendonça, T. et al. (2008) [33]. Microcracks were found in Groups 3 and 4. These results suggest that the intensive heat processing of sintering can damage the natural structure of the cancellous bone. Similarly, Gehrke et al. (2019) showed porous sintered bone structure. In another study, changes in bone morphology with gradually increased temperature were shown. At 120 °C, the water content was removed and the collagen fibrils became more evident. At 500 °C, almost all the polymer fibrils were burnt out, and only a few large size fibrils were retained. At 900 °C, hexagonal crystals were observed, and particles were converted into equiaxed polycrystalline particles. This phase transformation resulted in a lattice diffusion and morphology conversion between 750 °C and 900 °C [30]. De Carvalho et al. (2019) showed that higher sintering temperatures (820 °C and 1200 °C) resulted in a more grain-like architecture, which is similar to our crystalline structure at 1100 °C. Another study showed micro cracks, similar to our study, after 50 °C boiling for 4 h and sintering at 600 °C for 6 h. In this report, the bone samples had similar structural deterioration, such as the fissuring (microcracks) and chipping at the edge of pores seen in our study [35].

EDS analysis of the chemical composition of the samples revealed that the Ca/P ratio of Group 2 was higher than all other groups, even higher than the Ca/P ratio of certified HA (Ca_10_[PO_4_]_6_[OH]_2_) [40]. The higher Ca and P content in Groups 3 and 4 suggests that sintering resulted in loss of other components, which is similar to the work of Bi et al. (2010). They compared sintered bone to SCF-CO_2_–processed bone and found that Ca/P molar ratios of the bone samples from SCF-CO_2_ were similar to that of certified HA, with chemical elements in the typical range of bone tissue, including calcium, phosphorus, oxygen, carbon, magnesium, sodium and sulfur. In contrast, only calcium, phosphorus and carbon could be detected in sintered bone samples [35]. Another study analyzed the elements in the commercially available bone grafting materials Bio-Oss^®^ and Gen-Os (C, P, O and Ca), which were assumed to form a single phase of carbonated apatite with molar ratios of Ca:P ~1.65 and 1.60, respectively [40]. In our study, CaO and P_2_O_5_ in Group 4 (1100 °C) were 38.3% and 49%, respectively. Gehrke et al. (2019) demonstrated that their sintered bovine bone at 950 °C had 27.51 ± 5% and 25.66 ± 5 % of P_2_O_5_ and CaO, respectively. The biological apatite derived from bone products has a Ca/P ratio between 1.50 and 1.85. This ratio is strongly dependent on the bone species and the age of the animals [35]. An explanation for this stoichiometric deviation is the cationic and anionic substitutions of calcium, phosphate or hydroxyl groups from the hydroxyapatite lattice with trace elements and carbonate or silicate groups, respectively [41].

Work conducted in a rabbit model demonstrated that an increase in calcium and phosphate ions (Ca^2+^ and PO^43−^) provided by β-TCP created a desirable environment for the increase in proliferation and attachment of bone marrow–derived stem cells [42]. The release of Ca^2+^, PO^43−^ and HPO^42−^ from the material into the surrounding biological fluid provides nucleation sites for the precipitation of biological carbonated apatite. Moreover, the calcium and phosphate released from the β-TCP dissociation could increase osteoblast alkaline phosphatase activity. Previously, it has been proven that an increase in PO^43−^ in the surrounding medium provides an alkaline environment, which increases the alkaline phosphatase activity within human dental pulp cells and creates a conducive environment for osteogenesis [42]. On the other hand, a relatively high Ca/P ratio has been linked to delays in the resorption rate of HAp [37]. 

The American Society for Testing Materials (ASTM F-1839-08) considers the use of solid rigid polyurethane foam blocks as gold standard materials for the simulation of artificial bone in laboratory tests, as they present similar mechanical properties to human bone [43]. We chose to use Group 1 (raw bone) as a control and a wooden plank as a rigid support to represent the bone bed. Our simulated clinical drill testing reconfirmed the brittleness of the Group 3 bone blocks, with bone particles chipping off during drilling and fixation, this group also showed low mechanical strength during benchtop compression testing and demonstrated cracks in SEM analysis. A significant body of research has been conducted on the influence of drilling speed, axial drilling force and feed rate on bone drilling temperature [44]. In our study we maintained a constant drill speed of 800 rpm for all the samples to avoid variability. In this study, we conducted drilling and then placed the screw. Drilling for fixation can be done either in a single step or in multiple steps. In a single step, only one drill of the required diameter is used to produce the desired hole, whereas in multistep drilling, the drill diameter is gradually increased from the minimum to the required diameter using a number of drills [44]; in our case, two different sizes were used. Matthews et al. (1984) conducted experiments on human-cadaveric cortical bone to examine the effect of drilling of bone and found it a highly effective method for minimizing temperature elevation. It also gradually removes the material from the drilling site, resulting in less friction and better heat dissipation [45]. In the present study, since no data were available on sintered bone blocks’ ability to withstand clinical manipulation during screw placement, we preferred clinically oriented benchtop drill and screw placement tests over computational methods. This helped us predict the clinical outcome of these blocks upon manual manipulation. Following the recommendations of this paper, future computational studies can be designed to predict the best possible parameters of processing bone grafts to produce maximum strength of the bone blocks [46,47,48].

In vitro biocompatibility of the bone blocks was assessed using a metabolic assay and actin staining of HCOs. Group 4 graft material had the highest attachment at 24 h and metabolic activity for all time points as compared to the other groups. This could be due to the removal of most of the organic contents as confirmed by TGA, allowing the mineral surface of the bone to be more acceptable to the cells [49]. However, the reduction of proliferation at 48 h could be due to any of the cellular changes or variations in the microenvironment caused by the release of bone particles from the surface of sintered bone grafts, which could affect cellular response, as described in the study by Barbeck et al. (2015) [50]. HCO metabolic activity in Group 2 bone was higher than Group 3 bone throughout the 4 days; however, both showed consistently decreased metabolic activity after 48 h. This could be due to the removal of most of the organic contents (lipids and proteins) through boiling, while altering the surface structure with rough globular appearance as seen in SEM, making the surface more susceptible to cellular attachment. The surface composition and topographic effects (changes in surface roughness) significantly affected the osteoblastic activity, such as cellular attachment and bone forming ability [51]. An in vivo biocompatibility model study with minipig bone sintered at 820 °C and 1200 °C (30 min each) followed by sterilization in a dry oven at 120 °C showed significantly less bone regeneration than bone without sintering but with only chemical processing [33]. The higher-sintered bone had a significantly lower percentage of bone-to-material contact (BMC) when compared to the unsintered and 820 °C sintered bone groups. Moreover, both sintered bone groups showed a significantly lower percentage of new bone formation than chemically treated bone granules (particle size: 250–1000 µm) [51]. The author suggested this may be due to the crystalline changes during sintering, while chemical processing had no such changes. In a study by Aarthy et al. (2019), goat bone grafts produced by sintering at 900 °C and then 1100 °C, 1200 °C, 1300 °C and 1400 °C were tested with a human osteosarcoma cell line (MG63); the bone grafts were non-toxic to cell growth, and 1300 °C sintered bone grafts showed higher cellular proliferation [49]. On the contrary, a study comparing two sintered bone grafts (not block form), one at low temperature 300 °C (Bio-Oss^®^) and the other at a higher sintering temperature of 1250 °C (Bego-Oss^®^), found that higher heat treatment led to an increase in the inflammatory tissue response to the biomaterial as well as an increase in multinucleated giant cell formation [50]. This was ascribed to the embedding of bone substitute granules within the granulation tissue.

One limitation of our study is that we did not have a commercial product as a control. Though the bone graft produced in this study withstood the drilling process in the benchtop model, large animal in vivo models would help assess the bone block graft’s suitability for clinical application. Future testing and optimization of the resorption rate of bone block graft in either an in vitro or in vivo model is also important. As a bone scaffold, permeability is essential and is directly related to porosity and pore size [52]. Further testing of the effects of different treatments on porosity can provide greater insight regarding block graft’s role in regeneration. Fixation of the block graft as an onlay graft in the oral cavity will subject it to excessive loading stress due to masticatory forces. Therefore, future studies on block graft’s structural damage due to fatigue will be helpful in predicting its long-term success in in vivo settings [53].

The current sintered bone block grafts had almost no organic content, reducing any immunological concerns but at the same time significantly reducing strength as compared to raw bone. This could have potentially compromised the block graft’s ability to be screwed and maintain its structural integrity during bone fixation. However, from the mechanical testing, it became evident that higher temperature sintering was still able to significantly increase strength as compared to low temperature sintering, and drill testing confirmed its ability to withstand screw placement. Future processing techniques with lower temperature ranges or alternate non-thermal steps could potentially be adopted to preserve the mechanical strength of raw bone by overcoming the reduction of organic contents while simultaneous making the bone block grafts safer for use by removing potential immunological antigens.

Based on the findings of this study, sintered bone can be used to further develop bovine bone blocks as a graft after in vivo validation. However, extra care is needed when clinically handling the bone block grafts.

## 5. Conclusions

Sintering is an established processing method for bone graft development. However, to develop a bone ‘block’ graft with optimum physiochemical, mechanical and biological properties, sintering at two different temperatures was assessed using boiling followed by sintering. Bovine bone blocks sintered at higher temperatures (Group 4) resulted in highly pure bone with reduced organic components. Biocompatibility was higher for Group 4, as more osteoblasts were observed to be attached with higher proliferation. The mechanical strength and benchtop clinical handling of the higher sintered Group 4 was acceptable, as it withstood drilling and screw placement. Further animal model studies are required to demonstrate the suitability of this construct for bone grafting applications.

## Figures and Tables

**Table 1:** Thermogravimetric analysis of bone samples.

Groups	Treatments	Water	Organic Content	Residual Organic Content	Carbonate Decomposition
		%	%	%	%
1	Raw bone	8.11	17.55	11.87	3.03
2	Boil (6 h)	4.88	9.65	9.09	2.16
3	Boil (6 h), sintering 550 °C (6 h)	2.13	0.66	0.28	0.96
4	Boil (6 h), sintering 1100 °C (6 h)	0.02	0.02	0.02	0.000

**Table 2:** Percentage crystallinity of bone samples after different processing methods.

Groups	Treatments	Crystallinity (%)
1	Raw bone	23.31
2	Boil (6 h)	48.20
3	Boil (6 h), sintering 550 °C for 6 h	63.15
4	Boil (6 h), sintering 1100 °C for 6 h	95.33

**Table 3:** Chemical composition results obtained by energy dispersive X-ray spectroscopy.

Groups	1		2		3		4	
Elements	%	SD	%	SD	%	SD	%	SD
C	68.00	3.5	39.70	1.2	15.70	1.2	11.70	2.1
O	13.30	1.5	24.30	0.6	34.00	0.0	35.70	0.6
Na	0.40	0.1	0.30	0.0	0.20	0.2	0.00	0.0
Mg	0.20	0.1	0.40	0.1	0.60	0.2	0.40	0.3
Al	0.03	0.0	0.00	0.0	0.00	0.0	0.00	0.0
Ca	12.00	1.7	24.30	0.6	33.70	1.5	35.00	2.0
P	6.30	0.6	11.00	0.0	16.00	0.0	17.00	0.0
Ca/P ratio	1.90	0.1	2.20	0.1	2.10	0.1	2.10	0.1
P_2_O_5_	14.30	1.5	25.30	1.2	36.00	0.0	38.30	0.6
CaO	16.70	2.1	34.30	0.6	47.00	1.7	49.00	3.0

## Data Availability

The data presented in this study are available on request from the corresponding author. The data are not publicly available as they are part of a Ph.D. thesis and are available from the university library upon request.

## Supplementary Materials

The following supporting information can be downloaded at: https://www.mdpi.com/article/10.3390/bioengineering10040473/s1, Figure S1: Effect of different boiling durations on the mechanical strength of bovine cancellous bone with sintering at 1100 °C for three hours (*n* = 30). Mean ± SD, * *p* < 0.05; Table S1: Effect of different boiling durations on the mechanical strength of bovine cancellous bone with sintering at 1100 °C for three hours (*n* = 30); Figure S2: Effect of sintering at 1100 °C for different durations on the mechanical strength of bovine cancellous bone (*n* = 30). Mean ± SD, * *p*-value < 0.05; Table S2: Effect of different sintering durations on the mechanical strength of bovine cancellous bone after boiling (*n* = 30).
