# Supplementary figures and images for: Comparison of Low and High Temperature Sintering for Processing of Bovine Bone as Block Grafts for Oral Use: A Biological and Mechanical In Vitro Study
Source: Bioengineering (Basel). 2023 Apr 13;10(4):473. doi: 10.3390/bioengineering10040473 (PMC10136185; doi:10.3390/bioengineering10040473)

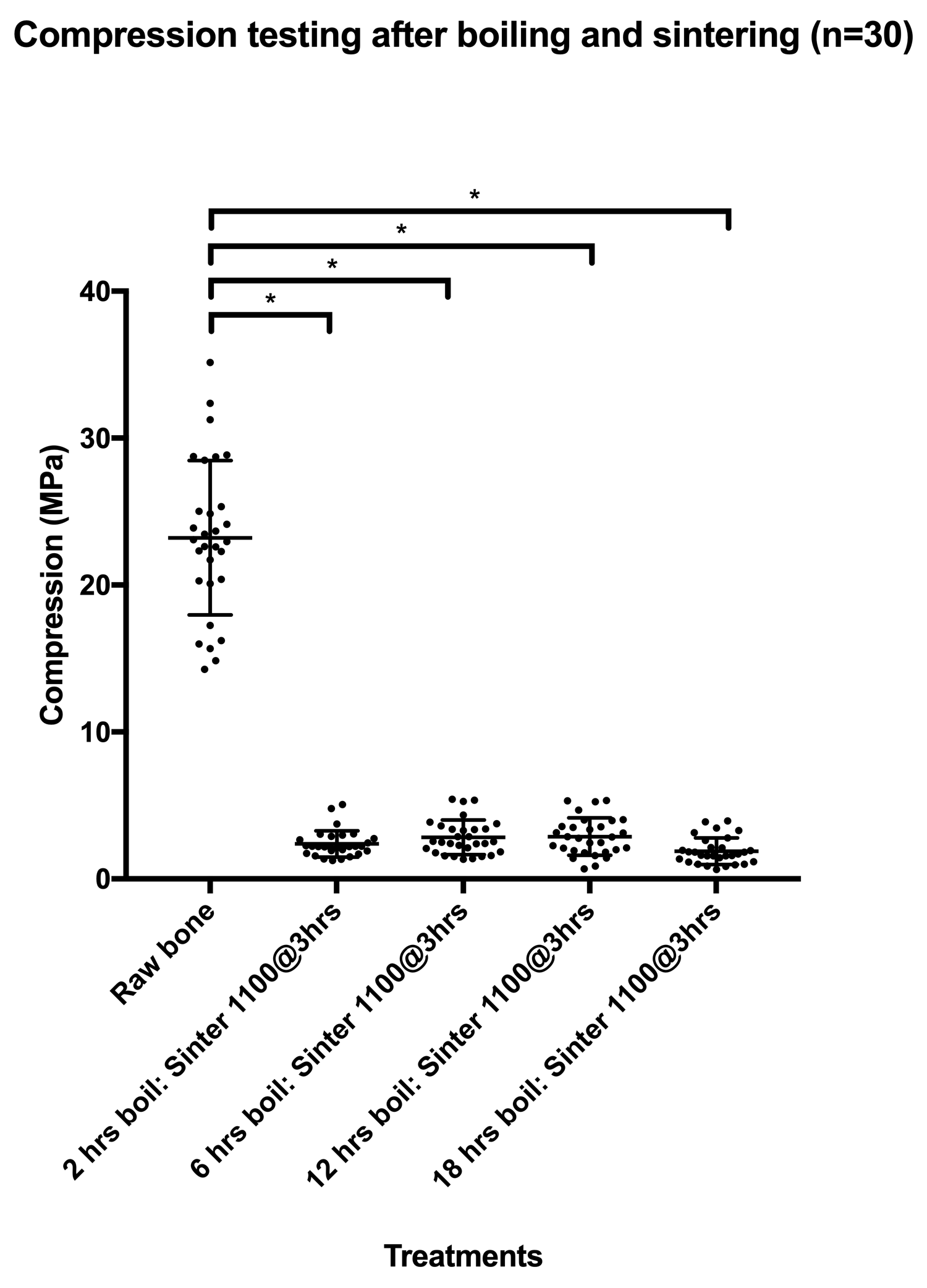

Supplement: Supplementary file 1 [file bioengineering-10-00473-s001.zip › Figure S1.tiff]

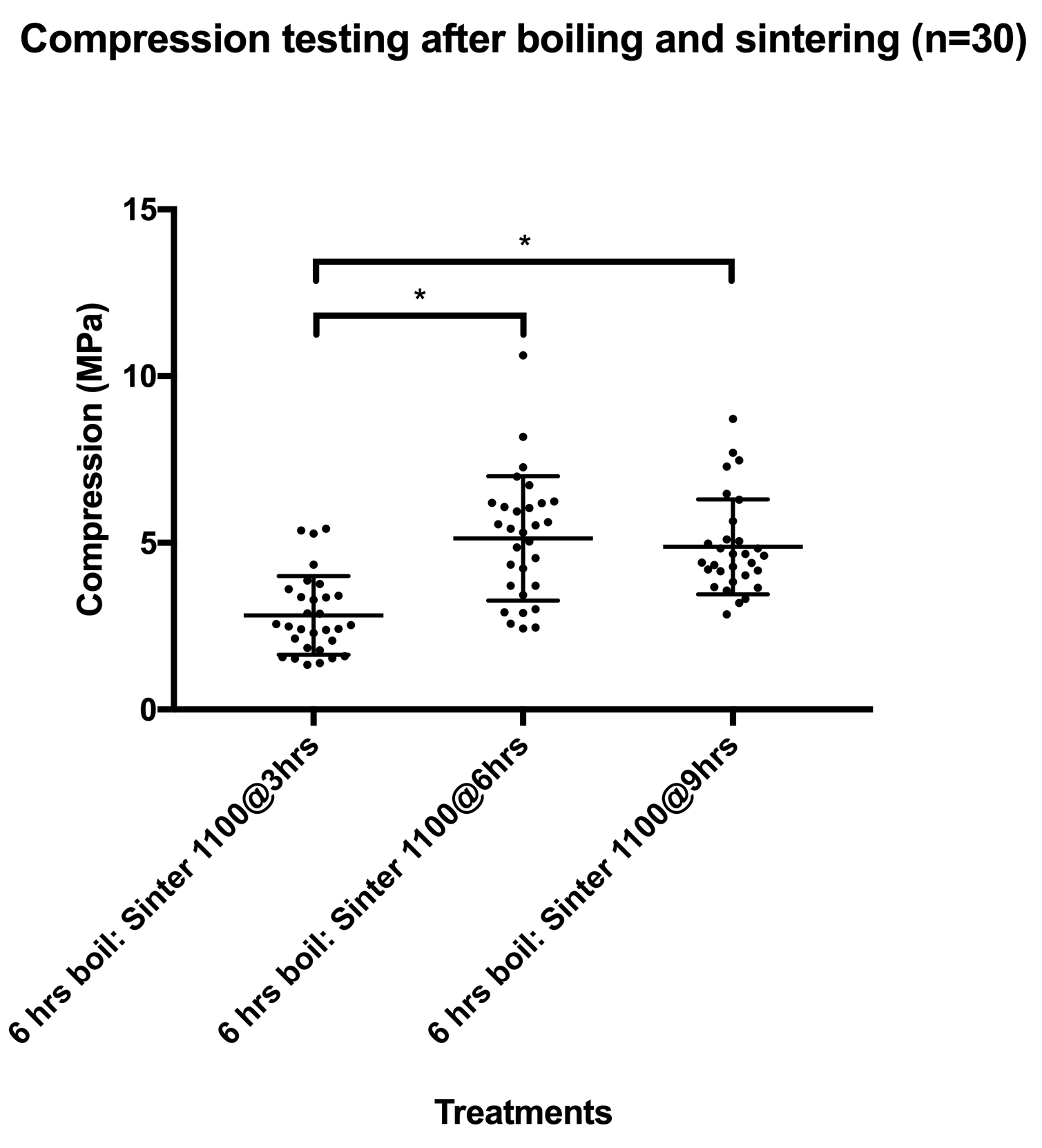

Supplement: Supplementary file 1 [file bioengineering-10-00473-s001.zip › Figure S2.tiff]
